# Opportunities in Development of Patient-Centric and Decentralized Clinical Trials: Insights from Patients and Healthcare Professionals in Respiratory and Rare Diseases

**DOI:** 10.1007/s43441-025-00819-6

**Published:** 2025-06-20

**Authors:** Marisa Minetti, Eva Topole, Ilaria Rondinone, Marta Lombardini, Paola Kruger, Mario Picozza, Simona Barbaglia, Andrea Terenzi, Ian Culverhouse, Lisa Forde, Isabella Montagna, Ylenia Paleari

**Affiliations:** 1 Chiesi Farmaceutici, S.P.A., Parma, Italy; 2 EUPATI, Rome, Italy; 3 FederAsma e Allergie ODV-Federazione Italiana Pazienti, Prato, Italy; 4 Associazione Nazionale Pazienti Respiriamo Insieme, Padua, Italy; 5 Doxa Pharma S.R.L., Milan, Italy; 6 Rebus Medical Ltd, Bristol, UK

**Keywords:** Decentralized clinical trial, Qualitative study, Respiratory disease, Rare disease, Patient perspective, Patient-centric, Direct-to-patient delivery

## Abstract

**Background:**

Decentralized clinical trials (DCTs) are increasingly being used to enable greater participation of patients in clinical research and improve the patient experience. In 2021, the Chiesi research and development team established the Digital innovAtion for patieNt Centric hEalth (DANCE) initiative with the aim to enhance the clinical trial journey for participants by merging patient perspectives with modern technology. As part of this project, the concept of DCTs was explored with patients affected by respiratory or rare diseases and with healthcare professionals (HCPs) treating these patients in the USA and Europe.

**Methods:**

The concept of the clinical trial journey and DCTs were initially explored through semi-structured interviews with 37 patients and HCPs. A follow-on web survey of 390 patients and HCP participants was then performed to gather opinions on the different components of DCTs and to identify elements to be incorporated in future DCTs. A final web survey with 135 patients and caregivers expanded on the DCT concept focusing on direct-to-patient delivery of investigational medications.

**Results:**

Overall, both patients and HCPs liked and were open to the concept of DCTs. They believed this approach in clinical trials would avoid patients having to travel long distances for screening or study visits, and increase participation of diverse populations in trials. The main concerns for both patients and HCP participants were the reduction of face-to-face interactions and whether the technology would be easy to use.

**Conclusions:**

This work highlights the benefits of DCTs and identifies potential challenges to be addressed to make DCTs more appealing to all participants. Responses also demonstrate the significance of integrating face-to-face contact with remote contact through user-friendly tools.

**Supplementary Information:**

The online version contains supplementary material available at 10.1007/s43441-025-00819-6.

## Introduction

In recent years, there has been an increase in the recognition of the importance of patients’ contributions to the design of clinical trial protocols, endpoints, and recruitment materials (often referred to as “patient centricity”) [1]. There is now acknowledgment that patients are experts about their conditions and therefore clinical development programs and trials should be designed with meaningful outcomes for patients while reducing patient burden. Furthermore, regulatory agencies such as the US Food and Drug Administration (FDA) and European Medicines Agency (EMA) recognize the importance of patient-oriented clinical development [2–4]. For example, the FDA holds patient-focused drug development meetings [2], and the EMA has a patient-inclusive approach to trial design engaging with patients through workshops [3, 5].

There is also growing interest in decentralized clinical trials (DCTs) due to their potential to reduce patient burden and enhance patient monitoring by remote collection of novel data sets, which support additional insights in disease natural history or treatment effects [3, 6]. DCTs are defined as clinical trials in which some or all the trial activities occur away from a traditional clinical trial site [6, 7]. Locations could instead include the home of the participant, a local healthcare facility, or a laboratory that is closer to the participant than the trial site [7].

These patient-oriented initiatives are driven by many considerations including the increased availability of digital health technologies (DHTs), which are systems that capture healthcare information directly from clinical trial participants [8, 9]. Many DHTs are portable instruments and can include devices that track activity, monitors for glucose levels or blood pressure, and spirometers (devices that measure lung function). These DHTs can be wearable devices, implants, or devices that are ingested [7, 8]. DHTs also include interactive mobile applications where participants can rate their quality of life, pain, depression, and daily functioning, or perform tests of functional performance such as cognition, coordination, and vision. The use of DHTs has reduced the need for visits to clinical trial sites and has increased focus on participant preferences when designing clinical trials.

The implementation of DCTs was accelerated during the coronavirus disease 2019 (COVID-19) pandemic in response to government-mandated restrictions, when the conduct of clinical trials needed to be modified to ensure the continuity of care [9, 10]. Since the COVID-19 pandemic, both the FDA and EMA have developed guidance on the implementation of DCTs [11–13]. DCTs are expected to reduce the need for clinic visits, consequently reducing participants’ travel time and costs, as well as improving recruitment and retention, and diversity, equity, and inclusion in clinical trials [9, 10, 14]. For example, participants who could not previously enrol in a traditional site-based trial (e.g., those living too far from the clinical trial site for regular visits, those unable to take time off work for regular appointments, or those unable to travel due to illness, lack of transport, or cost) may be able to participate in a DCT [7, 9, 10]. Taken together, these developments demonstrate that DCTs are feasible and acceptable to participants, thereby stimulating interest among patients, healthcare practitioners (HCPs), pharmaceutical companies, and regulatory bodies in exploring the potential of DCTs.

The Digital innovAtion for patieNt Centric hEalth (DANCE) initiative was established to enhance the clinical trial journey for participants, merging the patient perspective with modern technology through direct collaboration with the patient community and the adoption of innovative DHTs for remote data collection. It also planned to collect innovative datasets enabling novel, meaningful endpoints. To this aim, we explored the concept of DCTs with patients affected by respiratory or rare diseases, and with HCPs treating these patients in the USA and Europe. Rare and respiratory diseases were selected because they are the focus of the sponsor’s clinical development pipeline [15].

## Methods

### Project Design and Patients

**Fig. 1:**
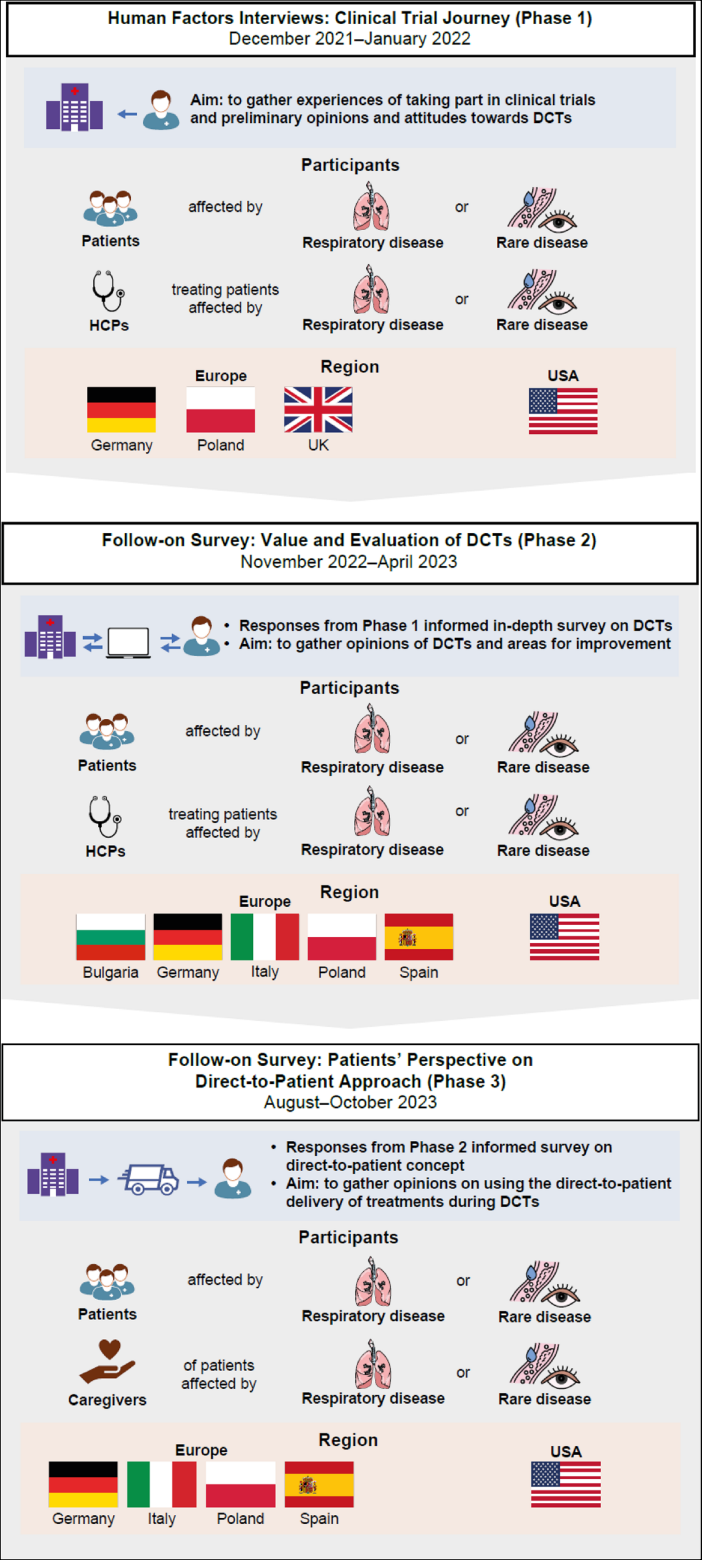
Project overview. *DCT* decentralized clinical trial, *HCP* healthcare professional

The project included three phases and employed a mixed methods approach with semi-structured interviews and two online surveys (Fig. 1). All participants provided written informed consent to participate in this project. The interview guide and the questionnaires were designed in collaboration with the patient community through input from patients and patient associations. The survey fell under the scope of market research and did not fall under the scope of regulations regarding clinical/observational studies or research involving humans. Therefore, submission to an ethical committee was not required. Special considerations were made to ensure the inclusion and safety of vulnerable populations, specifically individuals with respiratory and rare diseases. Participants were selected based on their ability to comfortably engage with the research methods, including phone interviews and online surveys. Phone interviewers were trained to be sensitive to the needs of participants with respiratory conditions, allowing for breaks or pacing if needed. For online surveys, the language was kept simple, and accessibility features such as larger text sizes and clear instructions were provided to accommodate participants with different needs. Additionally, both methods offered flexibility in terms of timing, allowing participants to choose convenient times to participate. The research was conducted with respect to privacy and confidentiality, and informed consent was obtained from all participants, ensuring that they understood the purpose of the research and any potential risks.

This research was conducted in accordance with the 1964 Declaration of Helsinki, and its later amendments, as well as British Healthcare Business Intelligence Association’s Legal & Ethical Guidelines for Market Research, the European Pharmaceutical Market Research Association, the European Society for Opinion and Market Research, and the Assirm Codes of Conduct.

#### Human Factors Interviews: Clinical Trial Journey (Phase 1)

An International Organization for Standardization (ISO) 13,485-certified specialist human factors consultant (Rebus Medical Ltd) performed semi-structured interviews. Interviews were conducted over video conference with the aim of understanding the experiences of patients and HCPs who have previously been involved with clinical trials and to explore their attitudes towards a decentralized approach to clinical trials. Patients affected by rare or respiratory disease [e.g., asthma, chronic obstructive pulmonary disease (COPD), idiopathic pulmonary fibrosis (IPF), chronic cough, Fabry disease, and adenosine deaminase deficient severe combined immunodeficiency (ADA-SCID)], and HCPs treating patients affected by these conditions were eligible for this study. Patient participants were required to be aged ≥ 18 years, have previously taken part in a clinical trial for their condition, and be willing to commit to a 60-min recorded session. HCPs were eligible if they had ≥ 5 years’ experience conducting clinical trials in patients affected by the conditions listed previously as either a principal investigator, clinical research coordinator/manager (or equivalent), or clinical trial nurse (or equivalent).

Between December 2021 and January 2022, participants from Germany, Poland, the UK, and USA were interviewed by a moderator in their local language using a semi-structured interview guide. These 60-min 1:1 virtual interviews were conducted at the participant’s place of residence. Topics covered during the interviews included experiences with study awareness and recruitment, the consenting process, provision or receipt of medication, disease monitoring and study visits, and the end of the clinical trial. The questions were tailored for each participant group (patient or HCP). Data analysis primarily focused on emerging themes. Descriptive statistics were used to describe patterns, trends, or frequency in data points across the sample using Microsoft Excel software. Not all participants responded to each question and some participants provided multiple responses to each question. Hence, the results are presented as follows: the numerator refers to the number of participants each who gave a specific response and the denominator is the number of responses received for each question.

At the end of the interview, if time allowed, participants were asked to take part in a scale exercise. Following the moderator describing the decentralized approach, for each stage of the clinical trial, patient participants were asked for each element described: “*If you were to take part in another clinical trial, using a scale from 1 to 10, where one is extremely unlikely and 10 is extremely likely, based on this description [of the decentralized approach for a specific element of the clinical trial], how much is this solution meeting your needs?”* Patient participants were also asked for each stage of the clinical trial process: *“Using a scale from 1 to 10, where one is no impact and 10 is high impact, how much do you think this solution would help/improve your life during the clinical trial?”* Reponses were presented as an average for all patient participants who gave a response.

#### Follow-on Survey: Value and Evaluation of DCTs (Phase 2)

Phase 2 was a follow-on survey, which commenced after completion of Phase 1; the results from Phase 1 were used to inform Phase 2. The objectives were to capture feedback to evaluate whether patients and HCPs were willing to participate in DCTs (and to what degree), to identify barriers to participation, and to find solutions to these barriers. Eligible participants were patients affected by respiratory or rare diseases (including asthma, COPD, IPF, chronic cough, bronchiectasis, pulmonary arterial hypertension [PAH], Fabry disease, and ADA-SCID) and HCPs treating patients affected by these conditions. Patients were required to be ≥ 18 years old. The eligibility criteria for HCPs were identical to those in Phase 1.

Between November 2022 and April 2023, participants from Europe (Bulgaria, Germany, Italy, Poland, and Spain) and the USA completed an online survey using computer-assisted web interview (CAWI) methodology (15 or 25 min for patients and HCPs, respectively). The online survey included closed and open-ended questions. Descriptive statistics were used to analyze responses. To determine significant differences, t-tests were used for means and z-tests for percentages [a p-value of 0.05 (95% confidence interval, CI) or 0.1 (90% CI) were considered as the thresholds for statistical significance].

#### Follow-on Survey: Patient’s Perspective on Direct-to-Patient Approach (Phase 3)

Phase 3 was designed to further explore the specific direct-to-patient approach used in the decentralization of clinical trials. The survey design was based on the results from Phase 2. The objective of Phase 3 was to collect feedback from patients about a new concept, known as a direct-to-patient service, for the delivery of investigational medication directly to the patient’s home. Eligible participants were patients affected by respiratory or rare diseases (including asthma, COPD, IPF, chronic cough, PAH, lysosomal storage disorders, endometabolic diseases, rare haematology and immunology diseases) and/or their caregivers. Participants were required to be aged ≥ 18 years and to have participated (or be a caregiver of a patient who participated) in a clinical trial in the 5 years before the survey was conducted.

Between August 2023 and October 2023, patients and/or their caregivers in Europe (Germany, Italy Poland, and Spain) and the USA completed an online survey of closed and open-ended questions (CAWI methodology). Descriptive statistics were used to analyze responses. To determine significant differences, t-tests were used for means and z-tests for percentages [a p-value of 0.05 (95% CI) or 0.1 (90% CI) were considered as the thresholds for statistical significance].

## Results

### Clinical Trial Journey (Phase 1)

Twenty-five patients and 12 HCPs participated in the 1:1 interviews in Phase 1. Baseline demographics are shown in Supplementary Table 1; over half of respondents were male and most patients were aged ≤ 60 years.

The interviews covered all aspects of the clinical trial process, from awareness and recruitment to the end of the trial. The first part of interview explored participants’ past experiences and the second half focused on their opinions towards a decentralized approach.

#### Study Awareness and Recruitment

Typically, participants were recruited into clinical trials through referral [*n* = 11/25 (11 patients responded they were recruited through referral and a total of 25 responses were received)], followed by newspaper advertisements, posters, or online methods (*n* = 7/25). Patients described the difficulties with travel as the main challenge during the recruitment process, including travel for pre-screening tests to determine eligibility and the lack of compensation for travelling (*n* = 4/9):

“If it was more local it would have been easier. I tried to get tests done locally, but it was difficult to get in contact with doctors [GPs] in […]. No cooperation between the local hospital and the study site.” UK P6 (COPD).

Other challenges for patients related to the impersonal methods of recruitment often used (*n* = 2/25):

“Would be good to do it over a video call such as skype, not just the telephone. More reassuring, I would feel better and would be able to see someone. When I got there, I didn’t know where to go, didn’t recognize anyone. Didn’t know if she [clinical trial staff who called beforehand] was a nurse? Would have been nice to see a familiar face.” Poland P4 (severe asthma).

Regarding the decentralized approach to recruitment, patients were positive about appointments/pre-screening being done remotely (*n* = 7/26), including saving time and money in avoiding hospital visits (*n* = 4/26), and the use of social media to receive information (*n* = 5/26). Patients’ main concerns were not wanting unfamiliar or specialist companies contacting them directly (*n* = 5/12), as generally patients preferred to be referred through a trusted source such as their specialist or a patient group:

“If it was someone, I didn’t know contacting me I probably wouldn’t participate. If it was someone who ran the foundation/association then I may participate. You need trust of the other party.” Poland P1 (severe asthma).

HCPs used a multi-pronged approach to maximize recruitment and the exact method depended on the trial requirements and the patient population. For HCPs, the main challenge around recruitment was finding suitable participants for clinical trials (*n* = 5/13). Regarding the decentralized approach to recruitment, HCPs reported a good level of response through social media, that tests conducted at the patient’s home allow them to learn about the patient ahead of time, and easier recruitment through decentralized methods (all *n* = 1/3). When asked about the perceived benefits and barriers for patients, the feedback aligned with the patients’ opinions. HCPs reported patients not needing to travel as a benefit (*n* = 1/2) and patients not wanting to be contacted by unfamiliar people/companies as they prefer to be approached by their doctor as a barrier (*n* = 2/10). Other barriers reported by the HCPs included needing funds to publish advertisements regularly (*n* = 1/10) and large numbers of ineligible patients applying for clinical trials (*n* = 1/10). One HCP reported:

“The initial onboarding conversation with the doctor or…study nurse is very important. It is important to create a trusting relationship, which is very imperative and necessary for the whole study. It means that data is then truthful. Human relationship is very important and often this is underestimated.” Germany HCP1.

#### Consenting Process

Patients consented mostly in person (*n* = 13/17), but signing at home or electronically were also mentioned (*n* = 4/17). Patients reported they found the documents not user-friendly (*n* = 6/16) as they did not always include the information they wanted to know (*n* = 4/16) and they would like more time to read the consent form (*n* = 2/16). Positive feedback on decentralized consenting included the ability to consent via video call (*n* = 10/45), sign electronically (*n* = 9/45), and save time due to not having to travel (*n* = 9/45); however, some patients would prefer in-person consenting (*n* = 4/12).

Current challenges reported by HCPs included making sure the patient understood fully before consenting and developing a relationship with the patient (*n* = 1/5 for both). They reported potential benefits of a decentralized approach to consenting, such as the use of an online platform (*n* = 2/3) and time saved (*n* = 1/3); for patients, they found the use of video calls as a benefit (*n* = 4/16). One HCP stated:

“Video call can be good for a patient who lives far away. If possible, it is better for the patient to have an actual face-to-face conversation.” Germany HCP1.

HCPs reported technology difficulties as a potential barrier (*n* = 4/12) and needing face-to-face discussions with the patient to ensure they fully understood the study (*n* = 3/12).

#### Provision or Receipt of Medication

Patient participants mainly collected their medication from the clinical trial site (*n* = 14/21). Current challenges in receiving medication included concerns about side effects/mental health (*n* = 6/18) and traveling to the clinical site for medication (*n* = 5/18). There were mixed views on the decentralized approach of medication delivery to the home. Some patients liked that they did not have to go to the hospital (*n* = 7/38) and liked the idea of home delivery (*n* = 6/38). However, some patients felt constrained having to stay at home for the delivery (*n* = 4/14).

For HCPs, current challenges included patients’ adherence to medication (*n* = 4/5) and lack of control (*n* = 3/5). For the decentralized approach, HCPs overall reported concerns regarding the lack of oversight on how and when patients took their medication, as well as concerns about other HCPs administering medication to their patients.

#### Disease Monitoring and Study Visits

Patients reported scheduling appointments and the impact on personal and work life as the current biggest challenges in the disease monitoring and study visits process (*n* = 13/20). Regarding feedback on a decentralized approach to study visits, patients liked completing the questionnaire/diaries by smartphone (*n* = 8/30), as well as the 24/7 virtual support (*n* = 6/30), and less frequent visits and less required traveling (*n* = 5/30). The main reported concern of patients centered on their ability to take certain measurements at home correctly without the aid of an HCP (*n* = 5/13).

For HCPs, the time-consuming nature of data entry and calculation was identified as a challenge from current experience (*n* = 2/12). HCPs did not perceive any benefits to the decentralized approach for HCPs; there were mixed feelings around whether HCPs believed patients could take measurements correctly at home (*n* = 3/7).

#### End of Study

Feedback from patients on the current challenges associated with the end of the study included an abrupt ending to the clinical trial (*n* = 2/6) and lack of/easy access to data (*n* = 3/6). One patient stated:

“Can be quite impersonal, treated in an impersonal way. At the end they just asked their last questions and just walked away. Could be improved. Didn’t ask if they could have improved my stay in the hospital. Could ask that question to make things easier.” UK P2 (mild asthma).

Patients reported several positive benefits to a decentralized approach to study closure, such as options to access data (*n* = 4/11), the facility to compare against others’ results (*n* = 4/11), video calls (*n* = 2/11), and access to data online (*n* = 1/11).

#### Scale Exercise

**Fig. 2:**
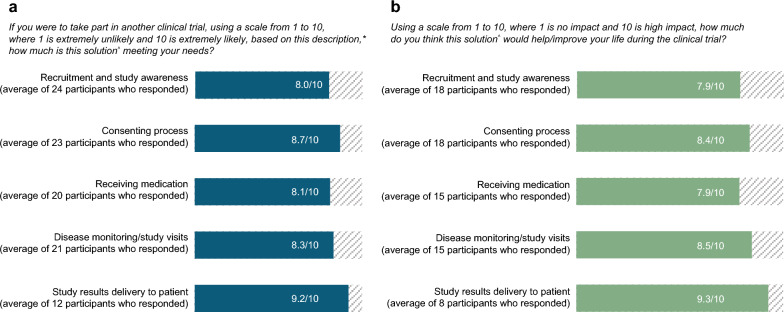
Token and scale exercise (Phase 1). **a** Token exercise. **b** Impact scale. * ‘*This description*’ refers to the description of the DCT approach. **^** ‘*This solution*’ refers to the DCT approach. *DCT* decentralized clinical trial, *HCP* healthcare professional

In the scale exercise, patient participants indicated that overall, the DCT approach meets their needs. Changes to the delivery of study results to the patient was the solution deemed to make them the most likely to take part in a future trial (Fig. 2a). When patients were asked to rate how much they thought the DCT approach would help/improve their life during a clinical trial, overall patients thought the approach would improve their lives (Fig. 2b). They rated delivery of study results to the patients as the change having the biggest impact.

Based on the responses around participants’ willingness to participate in and perceived benefits of the decentralized trial concept, a follow-on survey was developed to further explore the emerging themes.

### Value and Evaluation of DCTs (Phase 2)

A total of 206 HCPs and 184 patients participated in the survey. Across all regions, the majority of HCP and patient participants were male and most patient participants were aged ≤ 55 years (Supplementary Table 2).

The survey gathered participants’ responses on the concept of DCTs: what were their initial thoughts, did they see any advantages/disadvantages, and what were areas for improvement that would make DCTs more appealing or easier to participate in.

#### Opinions About DCTs

Overall, patients showed interest in and had a good impression of DCTs (62.5%), and this tended to be higher in the USA versus Europe and in rare diseases versus respiratory diseases (Table 1). Similarly, clinicians were positive about the concept of DCTs, with ≥ 64.0% considering them to be advantageous. Overall, HCPs had a higher intention to participate in a DCT (29.5%) compared with patients (27.1%).

**Table 1:** Opinions about decentralized trials (Phase 2)

Patient participants What is your first impression of these remote or decentralized clinical trials?						
	USA			Europe		
	Total	Respiratory	Rare diseases	Total	Respiratory	Rare diseases
Number of respondents	*n* = 50	*n* = 35	*n* = 15	*n* = 134	*n* = 122	*n* = 12
Positive comments	66%	63%	73%	61%	59%	75%
I like it/good impression/great/useful/I’d like to try	58%	57%	60%	45%	42%	67%
New/innovative	4%	3%	7%	5%	6%	–
Convenience	4%	3%	7%	3%	2%	8%
Easy recruitment of patients	2%	3%	–	–	–	–
Fast/quick methodologies	2%	–	7%	–	–	–
Easy to use/they make the visit easier	–	–	–	3%	2%	8%
No need to travel	2%	3%	–	3%	3%	–
Better disease management/monitoring	2%	3%	–	8%	8%	8%
Negative comments	12%	17%	–	17%	17%	17%
I don’t like/useless	4%	6%	–	11%	11%	8%
Poor quality data/privacy problems data	4%	6%	–	3%	3%	–
Impersonal/cold between doctor and patient	4%	6%	–	3%	3%	–
Technologies difficult to use/transmission problem	–	–	–	3%	2%	8%
Never tried/I need more information	22%	20%	27%	23%	25%	8%

HCP participants What is your general opinion about these remote or decentralized clinical trials?						
	USA			Europe		
	Total	Respiratory	Total	Respiratory	Total	Respiratory
Number of respondents	*n* = 80	*n* = 57	*n* = 23	*n* = 82	*n* = 63	*n* = 19
Positive comments	69%	70%	65%	66%	64%	74%
Good/interesting/advantageous idea	38%	39%	35%	43%	41%	47%
Good patient monitoring (better global assessment/to reach distant patients)	13%	16%	4%	7%	8%	5%
Convenience/easy recruitment of patients	8%	5%	13%	11%	8%	16%
Easy to use/easier and more effectiveness to work	8%	5%	13%	9%	6%	16%
Speed/saving time (fast methodologies that make the job faster)	–	–	–	2%	3%	–
Data/information quality	5%	7%	–	7%	8%	5%
Better data/collected data/accuracy	4%	5%	–	2%	3%	–
Better accessibility for patients to medical care	1%	2%	–	2%	3%	–
Good/automatization of data management/archiving data	–	–	–	2%	2%	5%
Negative comments	24%	21%	30%	29%	35%	11%
Don’t like these methods/I prefer face-to-face visit	10%	11%	9%	2%	3%	–
Poor convenience	5%	4%	9%	16%	19%	5%
Difficult to use also for patients, especially for older patients	4%	2%	9%	11%	14%	–
Difficult recruitment of patients	3%	2%	4%	–	–	–
Time consuming for use and training/work itself longer	–	–	–	5%	5%	5%
Data/information quality	5%	4%	9%	4%	3%	5%
Safety and problems of bias	3%	2%	4%	1%	2%	–
Too big data/poor quality info	1%	–	4%	2%	2%	5%
Bad data management	1%	2%	–	–	–	–
Other comments						
I have to try it/I need to gain experience	9%	11%	4%	11%	6%	26%
It depends on many factors/ it depends on type of study	3%	–	9%	2%	3%	–

*HCP* healthcare professionals

The results of the survey (Table 1) showed that HCPs in the USA believed DCTs allow for better patient monitoring, whereas HCPs in Europe rated convenience/easy recruitment of patients higher. HCPs in Europe expressed the potential difficulties for patients to participate in DCTs, especially for older patients, mainly due to challenges in using the DHTs, whereas this was less of a concern for US-based clinicians. A higher proportion of HCPs in the USA preferred face-to-face visits (10.0%) compared with HCPs in Europe (2.0%).

#### Main Benefits/Barriers for DCTs

Patients considered less travel and being at home to be the main advantages of, and motivations for, participating in DCTs. This was reported by a higher proportion of patients based in the USA versus Europe (Table 2). Benefits of DCTs rated higher in Europe versus the USA included saving time (with regard to administration of medicine) and money, and 24/7 virtual support, whilst remote visits and home deliveries/home visits were rated similarly between the two regions. Less travel was reported as a benefit by a significantly higher proportion of patients affected by respiratory diseases versus rare diseases within the USA [p = 0.036 (95% CI)]. Significantly more patients with respiratory diseases reported less travel as a benefit in the USA vs Europe [p = 0.033 (95% CI)]. Home deliveries/home visits were reported as benefits of DCTs with a numerically higher proportion of patients affected by respiratory diseases versus rare diseases in the USA. Whereas in Europe, home deliveries/home visits were reported by a significantly higher proportion of patients affected by rare diseases [p = 0.055 (90% CI)] versus respiratory diseases (Table 2).

**Table 2:** Main advantages/motivations and disadvantages/barriers of decentralized trials (Phase 2)

Patient participants						
	USA			Europe		
	Total	Respiratory	Rare diseases	Total	Respiratory	Rare diseases
Number of respondents	*n* = 50	*n* = 35	*n* = 15	*n* = 134	*n* = 122	*n* = 12
Main advantages If present, what could be the main advantages that these remote/decentralized clinical trials could have?						
Less travel	62%	71%*,† *p = 0.036 (95% CI) †p = 0.033 (95% CI)	40%* *p = 0.036 (95% CI)	50%	52%† †p = 0.033 (95% CI)	33%
Being at home	52%	51%	53%	32%	34%	25%
Saving time and money	40%	46%	27%	52%	52%	50%
Visits done remotely	26%	26%	27%	23%	23%	25%
24/7 virtual support	22%	23%	20%	33%	31%	42%
Home deliveries/home visits	18%	23%	7%	17%	14%* *p = 0.055 (90% CI)	42%* *p = 0.055 (90% CI)
Main difficulties If present, what are the main doubts that come to mind and what are the main problems that these remote/decentralized clinical trials could have?						
No in-person contact	34%	37%	27%	41%	45%	17%
Taking measurements without medical supervision	34%	37%	27%	30%	32%	17%
Concern about how data are collected, stored, and used	26%	29%	20%	27%	26%	33%
Need to be tech savvy	20%	14%	33%	27%	24%	50%
Don’t want to do tests at home/specific tests	16%	14%	20%	15%	15%	17%
Home assistant/HCP coming to your home	16%	17%	13%	13%	14%	8%
Don’t trust the delivery service	12%	9%	20%	6%	6%	8%
Don’t feel capable of handling necessary technology/activities	12%	14%	7%	21%	20%	25%

HCP participants						
Number of respondents	*n* = 80	*n* = 57	*n* = 23	*n* = 82	*n* = 63	*n* = 19
Main advantages For each of the following areas, what are, if any, the main benefits of these remote/decentralized clinical trials?						
Recruitment process/study awareness						
Patients don’t need to travel	83%	91%* *p = 0.006 (95% CI)	61%* *p = 0.006 (95% CI)	77%	79%	68%
Consenting methods						
Reduced travel-no travel costs/saves time	71%† †p = 0.032 (95% CI)	77%*,† *p = 0.088 (90% CI) †p = 0.006 (95% CI)	57%* *p = 0.088 (90% CI)	55%† †p = 0.032 (95% CI)	54%† †p = 0.006 (95% CI)	58%
Patient doesn’t need to come on site	59%	60%	57%	65%	65%	63%
Administration of medication						
Less clinic visits	59%	65%* *p = 0.083 (90% CI)	44%* *p = 0.083 (90% CI)	51%	59%* *p = 0.005 (95% CI)	26%* *p = 0.005 (95% CI)
Saves time	39%† †p = 0.094 (90% CI)	44%	26%† †p = 0.028 (95% CI)	52%† †p = 0.094 (90% CI)	51%	58%† †p = 0.028 (95% CI)
Study visits/disease monitoring						
Reduce the patients’ need to come to the clinical site	54%	56%	48%	62%	64%	58%
Main barriers For each of the following areas, what are, if any, the main limitations and problems of these remote/decentralized clinical trials?						
Recruitment process/study awareness						
Patient comfort and access to technology	56%† †p = 0.002 (95% CI)	60%† †p = 0.001 (95% CI)	48%	33%† †p = 0.002 (95% CI)	30%† †p = 0.001 (95% CI)	42%
Large numbers of ineligible patients applying for clinical trials	41%	44%	35%	32%	35%	21%
Difficult to get accurate pre-screening information from the patient	38%	42%	26%	44%	49%* *p = 0.053 (90% CI)	26%* *p = 0.053 (90% CI)
Sceptical about remote contact with patients	29%	28%	30%	37%	40%	26%
Consenting methods						
Technological barriers	59%† †p = 0.072 (90% CI)	68%*,†,‡ *p = 0.005 (95% CI) †p = 0.006 (95% CI)	35%* *p = 0.005 (95% CI)	45%† †p = 0.072 (90% CI)	44%† †p = 0.006 (95% CI)	47%
Need face-to-face discussion to ensure patients understand the study	34%† †p = 0.067 (90% CI)	33%	35%	48%† †p = 0.067 (90% CI)	48%	47%
Considerations for underserved/underrepresented communities	48%† †p = 0.002 (95% CI)	54%*,†,‡ *p = 0.039 (95% CI) †p = 0.013 (95% CI)	30%* *p = 0.039 (95% CI)	28%† †p = 0.002 (95% CI)	32%† †p = 0.013 (95% CI)	16%
Administration of medication						
Technological issues (system break, such as zoom)	41%† †p = 0.079 (90% CI)	44%	35%	28%† †p = 0.079 (90% CI)	29%	26%

To determine significant differences, t-tests were used for means and z-tests for percentages [a p-value of 0.05 (95% CI) or 0.1 (90% CI) were considered as the thresholds for statistical significance]

*HCP* healthcare professionals

*Statistical difference between groups in same region

†Statistical difference between corresponding group in other region

‡Statistical difference versus other group in same region and versus corresponding group in same region

The main barriers to DCTs reported by patients included taking measurements at home without medical supervision, concerns around data collection/storage/usage, and the need to be tech savvy; these were generally similar between patients based in the USA and Europe (Table 2). However, the lack of in-person contact was reported as a barrier by a higher proportion of patients in Europe versus the USA, the opposite of the trend seen in HCPs (Table 1).

When HCPs were asked what they believed to be the main advantages of DCTs, the top five reasons overall revolved around patients not needing to travel/reducing the need to travel during recruitment/study awareness, consenting, administration of medication, and study visit/disease monitoring stages (Table 2). This aligns with feedback from patient participants and this response was seen by a significantly higher proportion in HCPs treating patients affected by respiratory diseases versus rare diseases; again, aligning with the trend seen with the patient participants. The top five reasons were also generally consistent between HCPs in the USA and Europe, with some significant differences in the proportion of HCPs between the two regions (Table 2).

The top five difficulties of DCTs reported by HCPs differed between HCPs in the USA and Europe (Table 2). The same general themes seen in both regions were that technology was deemed to be a barrier during consenting/study awareness, consenting, and administration of medication. However, HCPs in Europe cited the lack of face-to-face discussions during consenting as the main difficulty, whereas HCPs in the USA cited technology barriers during consenting as their main perceived difficulty. These differences were significantly different between regions (Table 2). Of note, a significantly higher proportion of respiratory HCPs in the USA cited technological barriers [p = 0.006 (95% CI)] and considerations for underserved/underrepresented communities [p = 0.013 (95% CI)] as a perceived difficulty than their counterparts in Europe.

#### Suggestions for Improving DCT Approach

Suggestions for improving the DCT approach are shown in Table 3. When asked for suggestions for improving the approach of DCTs, HCPs identified a need for support for both the doctor and the patient (particularly in the USA) to make the system easier to use and to integrate with face-to-face visits. Patients considered the DCT approach as a great idea but 24% of patient respondents in the USA believe they should be financially compensated. Other feedback included that the DCT approach should be convenient (particularly in Europe) and should allow for good disease management.

**Table 3:** Suggestions for improving the model of decentralized clinical trials (Phase 2)

Patient participants						
Question	USA			Europe		
	Total	Respiratory	Rare diseases	Total	Respiratory	Rare diseases
Number of respondents	*n* = 50	*n* = 35	*n* = 15	*n* = 134	*n* = 122	*n* = 12
I like it/great idea, but they should be paid	24%	26%	20%	9%	9%	8%
For convenience	16%	20%	7%	22%	22%	25%
Easy to use/they make the visit easier	12%	17%	–	20%	19%	25%
Fast/quick/they make the visit faster	4%	3%	7%	4%	3%	8%
Allow good disease management	12%	14%	7%	18%	20%	8%
Patients followed at any time/to reach distant patients	12%	14%	7%	4%	4%	–
Allow a good/easy/quick relationship with the doctor	2%	3%	–	8%	8%	8%
Data/information quality	10%	9%	13%	16%	15%	25%
Safety/policy data	6%	6%	7%	5%	6%	–
Quality of info collected	2%	3%	–	5%	4%	8%
Accessibility/availability of info	2%	–	7%	7%	5%	17%
Focused on my disease	2%	3%	–	2%	2%	–
Social importance/ impact	–	–	–	1%	0%	8%
Improvement in quality of life	–	–	–	7%	8%	–
I don’t know	28%	20%	47%	27%	28%	25%

HCP participants						
Number of respondents	*n* = 80	*n* = 57	*n* = 23	*n* = 82	*n* = 63	*n* = 19
Support/help doctors-patients	23%	18%	22%	14%	14%	16%
Training courses for medical staff	9%	11%	4%	7%	6%	11%
More home healthcare nurses	6%	5%	9%	2%	3%	–
Training courses for patient	4%	2%	9%	5%	5%	5%
Convenience	14%	14%	13%	20%	18%	26%
Less complications/easier to use tools	6%	5%	9%	16%	13%	26%
Less parameters to be monitored	4%	5%	–	2%	3%	–
Simplify the part of technology used by patient	3%	4%	–	1%	2%	–
Fast/quick methodologies/techniques	1%	–	4%	1%	–	5%
Periodic face-to-face controls/visits	11%	11%	13%	6%	8%	–
Comments in general						
Better organization in general	8%	7%	9%	–	–	–
Implementation of these methodologies/more diffusion	6%	7%	4%	10%	11%	5%
Improvement of technology/working/operations	6%	9%	–	2%	3%	–
Standardization of all the protocols/processes	6%	9%	–	–	–	–
Don’t know	18%	18%	17%	26%	24%	32%

The results from Phase 2 showed the general openness of patients and HCPs to DCTs. Reduction in travel to the study site (for treatment and monitoring etc.) was frequently cited as an advantage of the DCT approach.

#### Treatment Acquisition and Administration

All patient participants (regardless of prior clinical trial experience) were asked questions regarding where their treatment was administered, where they picked up their medications, if home delivery services were offered by their treatment center, and how long they spent traveling to the doctor’s surgery or hospital center.

Overall, most patients never went to the doctor’s surgery or hospital center for treatment to be administered (USA 52%, Europe 56%), although a higher proportion of patients affected by rare diseases compared with respiratory diseases went to the hospital (occasionally or always; USA 60%, Europe 67%). Most patients went to the pharmacy to collect their medication (USA 60%, Europe 77%), and the proportion receiving their medication at home was significantly lower in patients in Europe (4%) versus the USA (32%). Centers in the USA were statistically significantly [p = 0.042 (95% CI)] more likely to offer a home delivery service compared with those in Europe (38% vs. 25%, respectively). A significantly higher proportion of patients in the USA [p = 0.012 (95% CI)] spent less than 30 min traveling to the doctor’s surgery/hospital center compared with those in Europe (78% vs. 60%, respectively).

Based on the results from Phase 2, a follow-on survey was developed to further explore the concept of direct-to-patient delivery of treatments, equipment for administering medications, and monitoring outcomes in DCTs.

### Patients’ Perspective on Direct-to-Patient Approach (Phase 3)

In total, 137 patients or their caregivers participated in Phase 3 (USA 50, Europe 87). Full baseline demographics are shown in Supplementary Table 3; ≥ 60% of participants were male and mean age was 42 years.

**Fig. 3:**
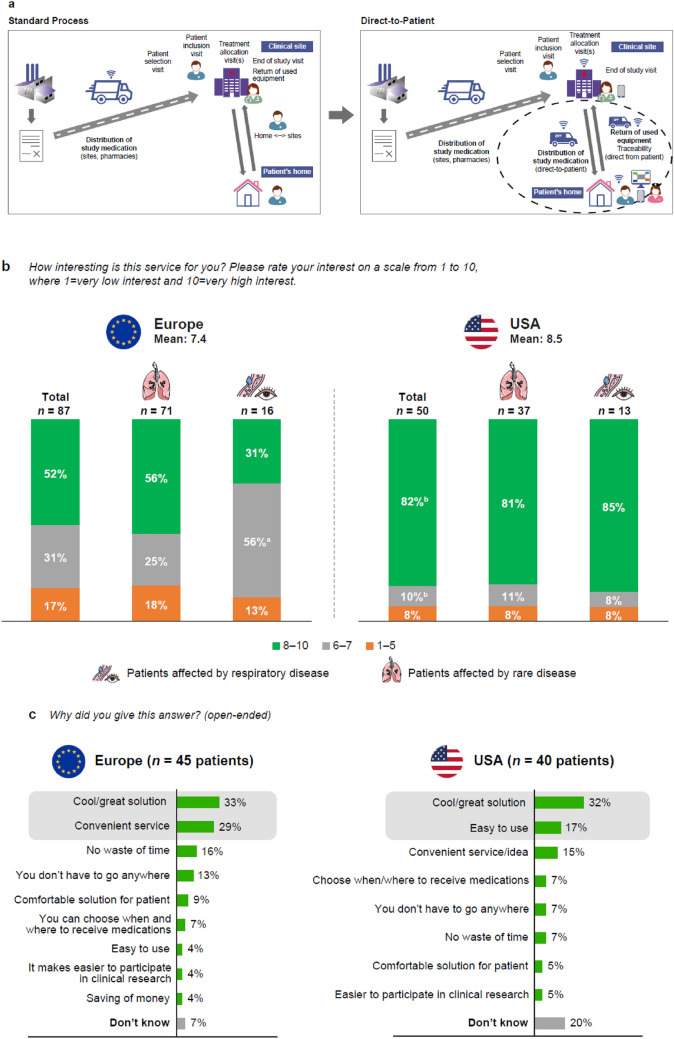
Direct-to-patient survey: patient’s perspective schematic (Phase 3). **a** Standard and direct-to-patient service. **b** Level of interest in the service. **c** Perceived benefits of the service

The direct-to-patient survey outlined the direct-to-patient approach to the participants and gathered their feedback. The current standard clinical trial process requires patients to travel to a clinical site for each study medication administration or to take the medication home for administration (Fig. 3a). A direct-to-patient service would allow clinical site pharmacies to deliver medication, equipment required for administration, and diagnostic tests directly to the patient’s home. After use, any used/unused medication and equipment are collected by the courier and sent back to the clinical site/pharmacy for final accountability and destruction (Fig. 3a).

Overall, the direct-to-patient service was met with high and uniform interest in both the USA and Europe (Fig. 3b); with the patient and caregiver participants agreeing the service is a great solution (Fig. 3c).

Most participants (81% USA, 80% Europe) did not have any concerns about the implementation of a direct-to-patient service but would prefer to have the possibility to choose a delivery time window to receive their medication (Table 4). In response to specific questions related to logistical aspects, participants expressed a range of preferred ways they would like to inform the clinical center about medication delivery. Most participants were interested in a pick-up service for used equipment, with significantly higher interest in the USA than in Europe [p = 0.005 (95% CI); Table 4]. Other preferences included returning the equipment personally or sending it by post. The majority of participants highly appreciated the optional services proposed because they met their need for flexibility (Table 4). Responses were generally consistent between the USA and Europe with the exception that participants in the USA were significantly more interested in being able to change the delivery time 2 days before the delivery compared with participants in Europe [interest of 8–10, p = 0.001 (95% CI); interesting of 6–7, p = 0.011 (95% CI)]. Participants showed a high level of interest for the availability of a tracking system for the shipment.

**Table 4:** Direct-to-patient survey (Phase 3): patients and caregivers

Question	USA			Europe			
	Total	Respiratory	Rare diseases	Total	Respiratory		Rare diseases
Would you prefer to receive the medicines directly at home?	*n* = 45	*n* = 33	*n* = 12	*n* = 84	*n* = 68		*n* = 16
Yes	84%	79%	100%	76%	73%		87%
No	16%	21%	–	24%	24%		13%
Would you like to have the possibility to define a delivery time window to receive the medicine?	*n* = 50	*n* = 37	*n* = 13	*n* = 87	*n* = 71		*n* = 16
Yes	84%	81%	92%	79%	77%		87%
No	21%	19%	8%	21%	23%		13%
Preferred time of delivery	*n* = 42	*n* = 30	*n* = 12	*n* = 69	*n* = 55		*n* = 14
In the afternoon from 1 to 5 PM	38%	33%	50%	55%	56%		50%
In the morning from 8 AM to 1 PM	38%	37%	42%	32%	29%		43%
Either in the morning or in the afternoon, but in a timeframe shorter than 3 h	21%	27%	8%	10%	11%		7%
Other time window	2%	3%	–	3%	4%		–
Preferred way to inform the clinical center about the delivery	*n* = 50	*n* = 37	*n* = 13	*n* = 87	*n* = 71		*n* = 16
Call the clinical trial center after the delivery	38%	41%	31%	41%	38%		56%
Text message to the clinical trial center with a picture showing the medicine	20%	24%	8%	30%	34%		13%
Video call to the clinical trial center after the delivery showing the medicine	14%	5%	39%	12%	10%		19%
Uploading the picture of the medication/supplies in a dedicated app or platform	10%	11%	8%	9%	9%		13%
QR code contained in the medical kit delivered to be sent to the site staff via phone or using the patient platform	16%	16%	15%	8%	10%		–
Pick-up service for used equipment level of interest (10 = high interest; 1 = low interest)	*n* = 50	*n* = 37	*n* = 13	*n* = 87	*n* = 71		*n* = 16
Mean	8.3			7.6			
8–10	80%* *p = 0.005 (95% CI)	76%	92%	58%* *p = 0.005 (95% CI)	59%	50%	
6–7	8%	11%	–	25%	25%	25%	
1–5	12%	14%	8%	17%	16%	25%	
Alternative ways to return used kits	*n* = 24			*n* = 7			
Prefer to return it personally	42%			–			
Mail/mail it myself/mail in a prepackaged container/deliver to the post office/deliver to head office/drop off at the parcel service	8%			n = 3			
Optional services: level of interest (10 = high interest; 1 = low interest)	*n* = 50			*n* = 87			
Designate a delegated person who can receive the medicine at the moment of the delivery	8–10: 70%			8–10: 62%			
	6–7: 16%			6–7: 25%			
	1–5: 14%			1–5: 13%			
Change the delivery time 1 day before the delivery	8–10: 66%			8–10: 55%			
	6–7: 24%			6–7: 26%			
	1–5: 10%			1–5: 18%			
Change the delivery time 2 days before the delivery	8–10: 78%* *p = 0.001 (95% CI)			8–10: 52%* *p = 0.001 (95% CI)			
	6–7: 12%* *p = 0.011 (95% CI)			6–7: 29%* *p = 0.011 (95% CI)			
	1–5: 10%			1–5: 20%			
Change the delivery location 2 days before the delivery	8–10: 58%			8–10: 51%			
	6–7: 20%			6–7: 22%			
	1–5: 22%			1–5: 28%			
Change the delivery location 1 day before the delivery	8–10: 58%			8–10: 49%			
	6–7: 18%			6–7: 25%			
	1–5: 24%			1–5: 25%			
Tracking system for the shipment: level of interest (10 = high interest; 1 = low interest)	*n* = 50	*n* = 37	*n* = 13	*n* = 87	*n* = 71	*n* = 16	
Mean	8.0			7.9			
8–10	72%	73%	72%	62%	65%	69%	
6–7	12%	8%	12%	24%	21%	23%	
1–5	16%	19%	16%	14%	14%	8%	

*Statistical differences between regions

To determine significant differences, t-tests were used for means and z-tests for percentages [a p-value of 0.05 (95% CI) or 0.1 (90% CI) were considered as the thresholds for statistical significance]

In Europe, some participants did not want their medications delivered at home as they would prefer to have direct contact/consultation with the doctor (25%), they were able to walk to the pharmacy near their home (20%), or that with delivery they need to stay at home and wait for the courier (15%). Several patient and caregiver participants in the USA stated: *“I worry about privacy and theft,”* and *“I wanted to consult with the dispensing pharmacist to be sure I had the right instruction and information to contribute effectively to the study.”* Most participants did not have any suggestions on how to improve the direct-to-patient service (USA 60%, Europe 75%); however, some participants suggested including the ability to contact the courier to arrange the delivery (Europe 7%), to provide a quality/accurate service (USA 10%), and to provide a functional/convenient service (USA 8%).

## Discussion

We found a high level of consistency between the patients and HCPs in their expectations of DCTs. This was especially evident for considerations such as the importance of face-to-face interactions and recognition of the benefits for patients in reducing the burden of traveling to clinical centers for appointments. Reducing the need for regular travel to the main clinical trial site for regular clinical visits could expand clinical trial access to include patients living further from major centers and therefore help improve recruitment into clinical trials, e.g., increasing participation from rural areas, poorer socioeconomic backgrounds, or a more diverse patient population. On the other hand, barriers to participation in DCTs can include being uncomfortable with wearable technology, concerns over data privacy, lack of access to technology, and lack of familiarity with technology.

There were consistent themes across the three phases, such as the concern among HCPs that patients will find the technology challenging (e.g., spirometry at home) or that HCPs at local clinics who are not familiar with the study may not take measurements or administer treatments correctly. Furthermore, we identified recurring themes such as the importance of trust between patients and HCPs. Participants preferred face-to-face contact with HCPs to build and maintain that trust. Previous studies have described how a trusting relationship between HCPs and trial participants is important for recruitment and retention of participants in clinical trials [10, 16].

In Phase 3 (direct-to-patient: patient’s perspective), it was identified that while patients liked the idea that they did not need to collect medications and supplies from the clinical trial site, they also appreciated the freedom and flexibility of the service together with the guarantee of quality. The role of the physician is vital to reassure patients on practical aspects of the service and making it as easy to use as possible and accessible to all patients, even those who are less familiar with technology. The feedback from this work will be used to determine the most appropriate direct-to-patient solution, to efficiently test the new process in a clinical trial and to minimize any potential patient concerns. This may include the option to deliver medications or supplies to the patient’s home, or delivery to a local medical facility for patients who want to maintain face-to-face contact.

In their systematic review of publications on DCTs, Rogers and colleagues identified four themes that are consistent with those identified in this work: research value, burden, safety, and equity [17]. The authors identified ease of participation, reduced burden of travel, and broadening access to clinical trials as benefits of DCTs, which reflects the findings of the current studies. Likewise, the authors of the systematic review identified better generalizability and representation of the real-world patient population as a benefit of DCTs [17]. Rogers and colleagues concluded that DCTs allow rapid development, but that there is insufficient evidence to confirm the most effective methods for trial recruitment and retention or the impact on cost of clinical trials. As in our results, Rogers and colleagues noted potential concerns about research quality in DCTs (e.g., lack of control over remote data collection and limited communications between researchers and patients) [17]. As in this project, the authors of the systematic review also identified potential concerns about confidentiality issues and trust.

In a paper published in 2023 on DCTs in rare diseases on behalf of the Drug Information Association Innovative Design Scientific Working Group [18], it was considered that DCTs offer a patient-centric approach that may help address equity concerns in clinical trials in rare diseases. The authors considered that the challenges of this approach include data privacy/security and the need to maintain patient engagement and retention [18]. There is a need to consider the specific requirements of the disease and quality of patient care, as well as the involvement of patients and caregivers in the clinical study design, including identifying which aspects of the study can be done remotely [18]. These findings reflect the benefits and concerns identified from patients and HCPs in the project.

Similar findings were also reported by the Trial Innovation Network in the USA, which has consulted on more than 400 research study proposals including DCTs [19]. The authors note that DCTs have the potential to improve diversity among participants (e.g., increasing participation from rural areas), but that there is a lack of data on inclusivity of racial and ethnic marginalized groups. The authors also mention concerns about online protections, digital access, and skills of study participants [19].

Due to the growing use and interest in DCTs, the US FDA, Department of Health and Human Services (DHSS), Center for Drug Evaluation and Research (CDER), Center for Biologics Evaluation and Research (CBER), Center for Devices and Radiological Health (CDRH), and Oncology Center of Excellence (OCE) issued joint guidance on the use of digital technologies in clinical trials in December 2023 [8], as well as draft guidance in May 2023 on conducting DCTs for drugs, biologics, and devices [6]. The guidance on the use of DHTs discusses opportunities to use digital technologies to collect data more frequently or continuously [8]. These guidance/guideline documents discuss how DCTs offer the potential to improve trial recruitment, and engagement and retention, and may also help involve participants with physical or cognitive impairments, or children. However, they highlight that DHTs have the potential to either expand or limit participation (e.g., excluding participants without digital skills) [8]. The draft guidance on DCTs considers all aspects of clinical trials including the conduct of trial, use of digital technology, participant consent, shipping of investigational products, safety, and use of software [6]. These guidelines link digital health developments with DCTs. The findings from our work show that patients and HCPs welcome the move towards DCTs and their benefits, such as improving retention and reducing the travel burden, but recognize that there are challenges in the uptake of DHTs.

In a paper published in 2024, the authors showed that the DCT structure enabled patients to self-test at home for respiratory viruses more frequently than patients without home-testing capability. Similar to the themes identified in our study, HCPs listed several concerns with DCTs such as low participant engagement, difficulty in ensuring that all participants understood the trials’ purpose, and challenges related to providing adequate instructions digitally. Another DCT published in 2025 investigating a digital therapy for pulmonary fibrosis found that the DCT structure was convenient for participants and allowed for a wide geographical outreach. However, the authors listed several drawbacks of the DCT structure, such as investigators not having access to full medical records/hospital notes, and the inability to perform physical examinations and computed tomography scans impeded the assessment of patient’s disease and potential adverse events.

### Study Strengths and Limitations

One of the strengths of this work is that it was conducted sequentially and the design of Phases 2 and 3 was informed by the design of the previous phases (i.e., Phase 1 informed the design of Phase 2, and Phase 2 informed the design of Phase 3). Whilst there has been much discussion about DCTs, here we have directly consulted patients and HCPs regarding their preferences and concerns about DCTs. In total, we involved more than 300 patients/caregivers and over 200 HCPs (primarily clinicians). Before this work, there was limited published research on patient and HCP experience of DCTs. For example, a recent systematic literature review of DCTs identified only 13 sources containing qualitative data on participant experience of DCTs [17]. Therefore, to our knowledge this project includes some of the largest qualitative/quantitative insights of patient and HCP attitudes to DCTs conducted to date.

The project focused on respiratory diseases and rare diseases (two therapeutic areas of interest for the sponsoring company) and the findings may not be applicable to other therapeutic areas. For example, the majority of patients enrolled had respiratory diseases and may be younger and fitter than participants with other diseases e.g., cancer, cardiovascular, or renal diseases. Participant age might influence preferences for traveling, the ability to travel, and familiarity with or the ability to use technology [7, 18].

## Conclusions

This project involving both patients and HCPs showed consistency of response between patients and HCPs, as well as identifying approaches to developing DCTs that address both patient and HCP needs and concerns. The findings from these studies highlight the importance of developing trust between HCPs and patients, understanding the needs of patients and their caregivers when participating in clinical trials, and the need for flexible approaches depending on the specific patient population and individual patient needs.

## Supplementary Information

Below is the link to the electronic supplementary material.

Supplementary file1 (DOCX 31 KB)

## Data Availability

Any data requests should be made to the corresponding author and will be considered by the authors.

## Abbreviations

ADA-SCID: Adenosine deaminase deficient severe combined immunodeficiency
CAWI: Computer-assisted web interview
CBER: Center for Biologics Evaluation and Research
CDER: Center for Drug Evaluation and Research
CDRH: Center for Devices and Radiological Health
COPD: Chronic obstructive pulmonary disease
COVID-19: Coronavirus disease 2019
DANCE: Digital innovAtion for patieNt Centric hEalth
DCT: Decentralized clinical trial
DHSS: Department of Health and Human Services
DHT: Digital health technology
EMA: European Medicines Agency
FDA: Food and Drug Administration
HCP: Healthcare professional
IPF: Idiopathic pulmonary fibrosis
ISO: International Organization for Standardization
OCE: Oncology Center of Excellence
PAH: Pulmonary arterial hypertension
SD: Standard deviation
