# Supplementary material for: Opportunities in Development of Patient-Centric and Decentralized Clinical Trials: Insights from Patients and Healthcare Professionals in Respiratory and Rare Diseases
Source: Ther Innov Regul Sci. 2025 Jun 20;59(6):1219–37. doi: 10.1007/s43441-025-00819-6 (PMC12579702; doi:10.1007/s43441-025-00819-6)
Supplement: Supplementary file 1 — Supplementary file1 (DOCX 31 KB) [file 43441_2025_819_MOESM1_ESM.docx]

**Supplementary tables**

# Table S1 Baseline demographics and clinical characteristics for human factors interview study: clinical trial journey (Phase 1)

| Characteristic, *n* (%)* | Participants in Europe (*n*=28) | | Participants in the USA (*n*=9) | |
| --- | --- | --- | --- | --- |
|  | Patients (*n*=18) | HCPS (*n*=10) | Patients (*n*=7) | HCPS (*n*=2) |
| Age, years |  |  |  |  |
| ≤60 | 8 (44) | – | 1 (14) | – |
| ≥61 | 2 (11) | – | 1 (14) | – |
| Data not available | 8 (44) | 10 (100) | 5 (71) | 2 (100) |
| Gender^†^ |  |  |  |  |
| Male | 9 (50) | 5 (50) | 4 (57) | – |
| Female | 9 (50) | 5 (50) | 1 (14) | 2 (100) |
| Indication |  |  |  |  |
| Respiratory disease | 17 (94) | 10 (100) | 1 (14) | 1 (50) |
| Rare disease | 1 (6) | 0 | 6 (86) | 1 (50) |

*Unless otherwise stated. Data may not add up to 100% due to rounding. ^†^One patient participant in the USA responded as non-binary, and one did not disclose.

SD=standard deviation.

# Table S2 Baseline demographics and clinical characteristics for follow-on survey: value and evaluation of DCTs (Phase 2)

| Characteristic (%)* | Participants in Europe | | Participants in the USA | |
| --- | --- | --- | --- | --- |
|  | Patients (*n*=134) | HCPs (*n*=106) | Patients  (*n*=50) | HCPs (*n*=100) |
| Age, years |  |  |  |  |
| ≤40 | 37 | 34 | 36 | 28 |
| 41-55 | 36 | 41 | 32 | 51 |
| ≥55 | 27 | 26 | 32 | 21 |
| Gender^†^ |  |  |  |  |
| Male | 48 | 59 | 42 | 75 |
| Female | 52 | 37 | 56 | 20 |
| Area of residence |  |  |  |  |
| Large city | 55 | 77 | 36 | 61 |
| Small city/town | 15 | 8 | 40 | 30 |
| Suburb near a large city | 26 | 14 | 6 | - |
| Rural area | 5 | 1 | 18 | 9 |
| Indication, *n* (%) |  |  |  |  |
| Respiratory disease | 122 (91) | 76 (72) | 35 (70) | 70 (70) |
| Rare disease | 12 (9) | 30 (28) | 15 (30) | 30 (30) |

*Unless otherwise stated. ^†^Prefer not to disclose: Europe: 4% HCPs; USA: 2% patients, 5% HCPs.

DCT=decentralized trial; HCP=healthcare professional; SD=standard deviation.

# Table S3 Baseline demographics and clinical characteristics for follow-on survey: patient’s perspective on direct-to-patient approach (Phase 3)

| Characteristic, (%)* | Participants^†^ in Europe (*n*=87) | Participants^†^ in the USA (*n*=50) |
| --- | --- | --- |
| Age, years |  |  |
| Mean | 42.3 | 42.0 |
| ≤18^‡^ | 2 | 6 |
| 19–25 | 15 | 10 |
| 26–35 | 25 | 24 |
| 36–45 | 18 | 28 |
| 46–54 | 15 | 8 |
| ≥55 | 24 | 24 |
| Gender |  |  |
| Male | 60 | 64 |
| Female | 40 | 46 |
| Employed | 69 | 76 |
| Area of residence |  |  |
| Large city | 52 | 60 |
| Small city/town | 26 | 16 |
| Suburb near a large city | 20 | 14 |
| Rural area | 2 | 10 |
| Level of education |  |  |
| Degree | 46 | 52 |
| High school | 45 | 42 |
| Middle or elementary school | 9 | 6 |
| Indication, *n* (%) |  |  |
| Respiratory disease | 71 (82) | 37 (74) |
| Rare disease | 16 (18) | 13 (26) |

*Unless otherwise stated. ^†^Patient with respiratory or rare disease or caregiver of patient with respiratory or rare disease. ^‡^Participants for these patients were caregivers.

SD=standard deviation.
